# A Novel Catheter-Based Method for Denervation of Afferent Renal Nerves in Sheep

**DOI:** 10.1007/s13239-025-00786-x

**Published:** 2025-05-06

**Authors:** Arthur de la Cruz-Lynch, Brianna Dailey-Krempel, Alex Dayton, Duc T. Nguyen, Roman Tyshynsky, Dusty Van Helden, Matthew Lahti, John Carney, Louise Evans, Lucy Vulchanova, John Osborn

**Affiliations:** 1 Department of Surgery, University of Minnesota, Minneapolis, MN 55455 USA; 2 Department of Integrative Biology & Physiology, University of Minnesota, Minneapolis, MN 55455 USA; 3 Medical Scientist Training Program, University of Minnesota, Minneapolis, MN 55455 USA; 4 Department of Neuroscience, University of Minnesota, Minneapolis, MN 55455 USA; 5 Experimental Surgical Services Laboratory, University of Minnesota, Minneapolis, MN 55455 USA

**Keywords:** Catheter based, Renal, Denervation, Afferent renal denervation, Sheep, Peregrine

## Abstract

**Purpose:**

Catheter-based total renal denervation (TRDN) has recently gained FDA approval to lower blood pressure in patients with treatment-resistant hypertension. Current TRDN technologies indiscriminately destroy efferent (sympathetic) and afferent (sensory) renal nerves. However, preclinical studies suggest that the beneficial effects of TRDN may be due to ablation of afferent, rather than efferent, renal nerves. We developed a novel method for chemical ablation of afferent renal nerves by periaxonal application of capsaicin which has been employed in mouse and rat models of hypertension. In certain rodent models afferent-specific renal denervation (ARDN) has been shown to lower arterial pressure to the same degree as TRDN. The objective of the present study was to develop a catheter-based method for ARDN in a large animal model with the long-term goal of translating this treatment to humans. We tested the feasibility of using the Peregrine™ catheter infusion system, currently used to perform TRDN in humans by injection of ethanol, to perform catheter-based afferent renal denervation in sheep by periaxonal application of capsaicin.

**Methods:**

Castrated, adult, male, Friesen sheep underwent Sham RDN (saline, *n* = 2), TRDN (ethanol, *n* = 4), or ARDN (capsaicin, *n* = 4) with the Peregrine™ catheter before termination > 2 weeks after the procedure. Denervation of renal efferents was verified by measurement of renal cortical norepinephrine (NE) content and anti-tyrosine hydroxylase (TH) staining; denervation of renal afferents was verified with anti-calcitonin gene-related peptide (CGRP) staining.

**Results:**

There was a significant decrease in TH + and CGRP + fibers in TRDN kidneys and in CGRP + but not TH + fibers in ARDN kidneys. TRDN significantly reduced renal cortical norepinephrine (NE) content by 89% while ARDN had similar NE content to Sham RDN kidneys.

**Conclusions:**

This study establishes the feasibility of performing catheter-based afferent renal denervation in a large animal model. Furthermore, this study provides a translational model to evaluate catheter-based ARDN as a potential treatment for hypertension.

## Introduction

Recent clinical trials utilizing three different renal denervation catheters have reported that catheter-based total (efferent and afferent) renal denervation (TRDN) effectively lowers arterial pressure in patients with treatment-resistant hypertension (HTN) [1,2,3]. These catheters work by delivering heat or neurolytic chemicals from the renal artery lumen to destroy renal nerves that travel along the renal artery media and adventitia. Two of these catheters, Medtronic’s Spyral™ radiofrequency-based renal denervation system and Recor Medical’s Paradise^®^ Ultrasound-based renal denervation system, have gained FDA approval for use in treatment-resistant HTN [4,5]. The third catheter, Ablative solutions’ Peregrine System™ Infusion Catheter, just reached its sham-controlled phase III clinical trial’s primary endpoint by demonstrating a statistically significant difference in 24-hour ambulatory blood pressure three months after the procedure [6].

The conventional explanation for how renal denervation lowers blood pressure is by ablation of the efferent (sympathetic) renal nerves which transmit input from the brain to the kidney to regulate renal blood flow, sodium reabsorption, and renin release [7]. However, the kidneys are also innervated by afferent (sensory) nerves, which project to circuits in the spinal cord and brain that modulate sympathetic output to peripheral targets [8,9,10]. Interestingly, clinical trials reported several unintentional, yet beneficial side effects following renal denervation which included improved glucose metabolism [11], decreased incidence of cardiac arrhythmias including atrial fibrillation [12], decreased muscle sympathetic nerve activity [1, 13], and decreased frequency of sleep apneic episodes [14]. These findings are not directly related to disruption of efferent renal nerve function, so they are thought to be a result of interrupting sensory renal nerve feedback to brain regions which modulate sympathetic output to various organ systems that could contribute to cardiometabolic disease. Efferent (sympathetic) and afferent (sensory) renal nerves are intermixed in the same nerve bundles [15], and current catheter-based TRDN technologies destroy both types of nerves indiscriminately.

To investigate the role of afferent renal nerves in HTN, our lab developed a novel method of afferent-specific renal denervation (ARDN) via periaxonal capsaicin application in the rat [16]. Capsaicin is a potent agonist for the TRPV1 channel, which is present in afferent, but not efferent, renal nerves. Sustained activation of this channel results in a massive calcium influx resulting in nerve fiber destruction. Using this method, our group and others have shown that ARDN attenuates the progression of HTN to the same degree as TRDN in DOCA salt rats [17], DOCA-salt mice [18], 2 kidney, 1 clip (2K1C) rats [19], and 2K1C mice [20]. These results suggest that the effects of TRDN on arterial pressure were due to ablation of afferent, rather than efferent, renal nerves.

Despite the excellent safety profile of catheter-based TRDN, studies in sheep demonstrated that this method impaired the ability to maintain arterial pressure during hemorrhagic or septic shock [21,22], presumably due to the loss of efferent renal nerve control of renal vascular resistance, renin release, and sodium reabsorption. These observations, combined with evidence that ablation of afferent renal nerves mediates the response to TRDN, supports the need to develop a catheter-based method for ARDN in humans. With that goal in mind, we used the Peregrine Catheter Infusion System™, which deploys microneedles from the renal artery lumen to inject ethanol, a neurotoxin, into the renal artery adventitia [3], to determine the feasibility of periaxonal administration of capsaicin (instead of ethanol) to selectively destroy afferent renal nerves, while sparing efferent renal nerves in a large animal model.

## Materials and Methods

### Animals

All sheep used in this study received care based on protocols approved by the University of Minnesota’s Institutional Animal Care and Use Committee based on guidelines for humane animal care. All procedures were performed by trained experimental surgical staff from Experimental Surgical Services, a core facility at the University of Minnesota Department of Surgery.

10, castrated-male, Friesen/Friesen cross sheep (Weight: 60–80 kg; Age: 1–2 years) were used for this study. Four sheep were used for catheter-based total renal denervation (TRDN), four sheep were used for catheter-based afferent renal denervation (ARDN), and two sheep were used as Sham denervated (Sham RDN) controls.

### Capsaicin Preparation

The capsaicin dosage was based on previous sensory denervation studies in the adrenal medulla [23,24,25], and the solution was used to safely and effectively perform afferent renal denervation in rat and mouse studies [16,17,17,18,19,26]. Capsaicin (Sigma Aldrich, Catalog #M2028) was first dissolved in absolute ethanol (Fisher BioReagents, Catalog# BP2818100). Then Tween 80 (Sigma Aldrich, Catalog #P1754) and normal saline (Baxter, Catalog# 2F7123) are added such that final concentrations were 33 mM capsaicin, 5% ethanol, 5% Tween 80, and 90% normal saline. This capsaicin solution was then vigorously mixed immediately before being drawn up through a 1 mL luer-lock syringe and injected through the Peregrine™ catheter.

### Renal Denervation Treatments with the Peregrine Catheter Infusion System

An intramuscular injection of Ketamine (10 mg/kg) and atropine (0.04 mg/kg) was given for sedation and to reduce salivary secretions, respectively. After aseptic preparation, a catheter was percutaneously placed in a peripheral vein (jugular or medial saphenous) for intravenous (IV) access. An IV injection of Propofol (2–6 mg/kg) was given to induce anesthesia before intubation with an endotracheal tube. An orogastric tube was then placed and ~ 500 mLs of Magnalox (Sparhawk Laboratories, Inc, Catalog# M-0737-09), a mild laxative/antacid solution, was administered via the orogastric tube to prevent bloating and acidosis post-operatively. Both inguinal regions were then shaved in preparation for surgery.

The animal was then secured in a supine position on a heated operating table. Mechanical ventilation was then initiated at 10–15 breaths per minute. Oxygen was set at approximately 3–4 L/min, and isoflurane (Piramel Pharma Limited, NDC 66794-017-10) was set between 1 and 4% as needed to maintain a deep plane of anesthesia. The animal was considered deeply anesthetized when the eye and/or jaw reflex was absent. ECG monitoring leads were then positioned on the sheep’s front left, front right, and rear right limbs. End tidal CO2 (ETCO2) and saturated O2 (SpO2) were monitored, and the sheep’s baseline vital parameters were recorded once the sheep was stable. Bilateral inguinal regions were then scrubbed before the entire animal, except for the inguinal regions, was draped in preparation for sterile surgery.

**Fig. 1:**
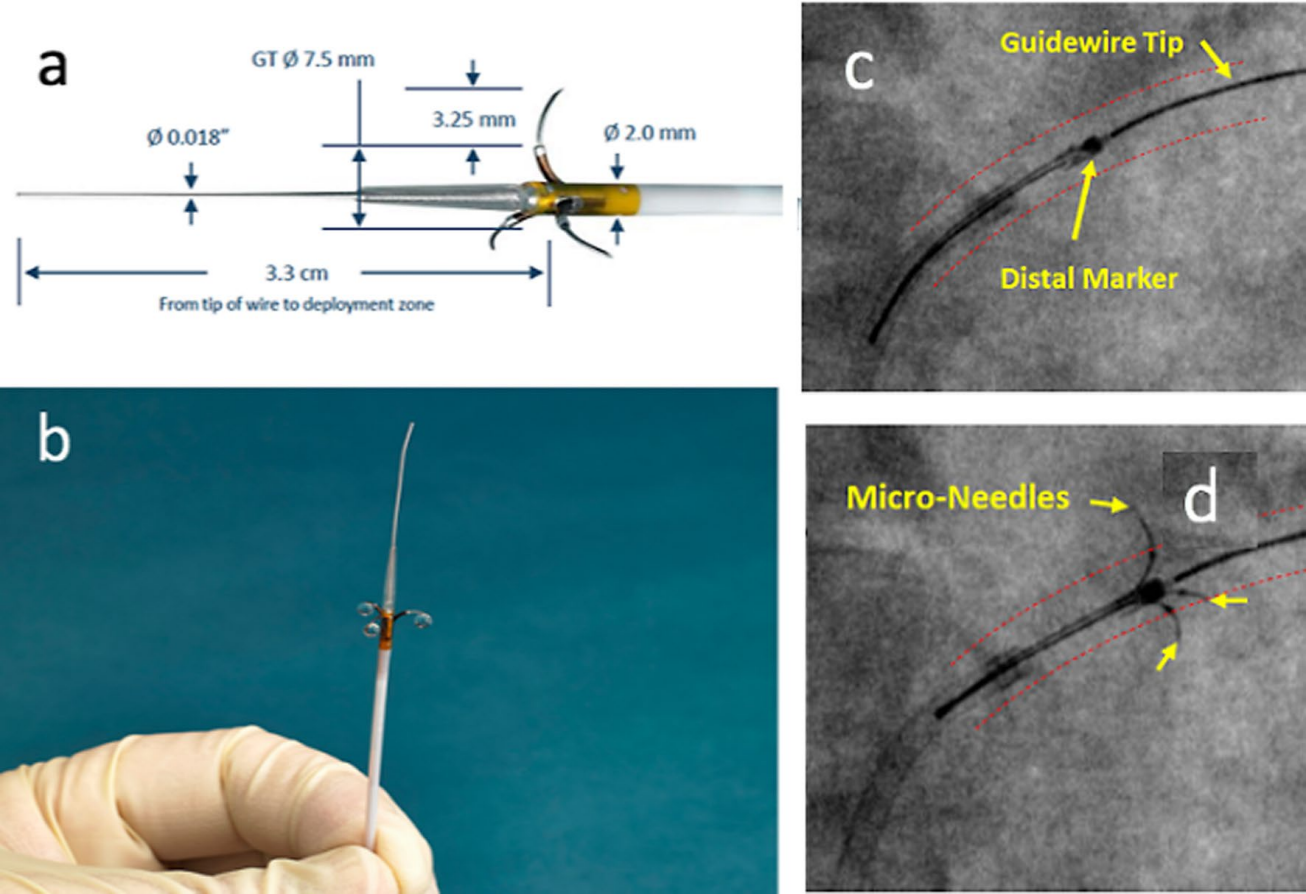
Peregrine catheter infusion system dimensions. (**a**) of the Peregrine™ catheter tip. Image (**b**) of the Peregrine™ catheter outside the body during alcohol infusion. Fluoroscopic images of the Peregrine™ catheter before (**c**) and after deployment of the guide tubes and microneedles in a renal artery. This figure was obtained from images in a previous publication [3]

A cut-down was then performed to access the right femoral artery. After the initial incision with a scalpel, sterile surgical instruments and cautery were used to dissect tissue and provide hemostasis. Vessel loops were double looped at the proximal and distal segments of the common femoral artery for hemostatic control. An 8 French sheath was utilized for femoral arterial access, and an appropriately shaped angiographic guide catheter was then advanced over a 0.035-inch J-tipped guidewire into the abdominal aorta to the level of the renal arteries using fluoroscopic guidance. The angiographic guide catheter was then used to enter the renal artery and then 5–8 ml of a radiopaque diagnostic contrast agent was injected during live fluoroscopic imaging (baseline renal artery cineangiography). The Peregrine™ catheter infusion system (Fig. 1) was prepped by flushing with heparinized saline. The main ports were flushed first, then the hypo/guide tubes were deployed and flushed. Afterwards, the microneedles were deployed and flushed. Careful attention was paid to verify that heparinized saline was coming out of all three microneedles. After verifying that the needles were patent, the Peregrine™ catheter was then advanced into the renal artery via the angiographic guide catheter. After advancing the catheter halfway through the length of the renal artery, the hypo/guide tubes and then the microneedles were deployed. Fluoroscopy was used to verify that the catheter was centered in the renal artery lumen. While clinical trials [27] for the Peregrine catheter infused 0.6 mL of ethanol to achieve total renal denervation, our study infused 1 mL of ethanol, capsaicin, or saline per renal artery. This infusion volume was chosen because it was well tolerated in preliminary studies and allowed for sufficient volume to surround the renal vessels completely. The microneedles and hypo tubes were retracted, and the catheter was then removed. At least 2 min after each injection, 5–8 mLs of a radiopaque diagnostic contrast agent was injected through the angiographic guide catheter to assess for vessel injury. Occasionally, a small amount of leakage into the renal artery adventitia would occur but would disappear after ~ 5 min. After following the same procedure to treat the other kidney, the needles and hypo tubes were then retracted, and the Peregrine catheter was removed before a final angiogram was performed to verify vessel integrity. The femoral artery was repaired and the incisions were closed using standard techniques as previously described [28,29].

### Termination and Tissue Collection

**Fig. 2:**
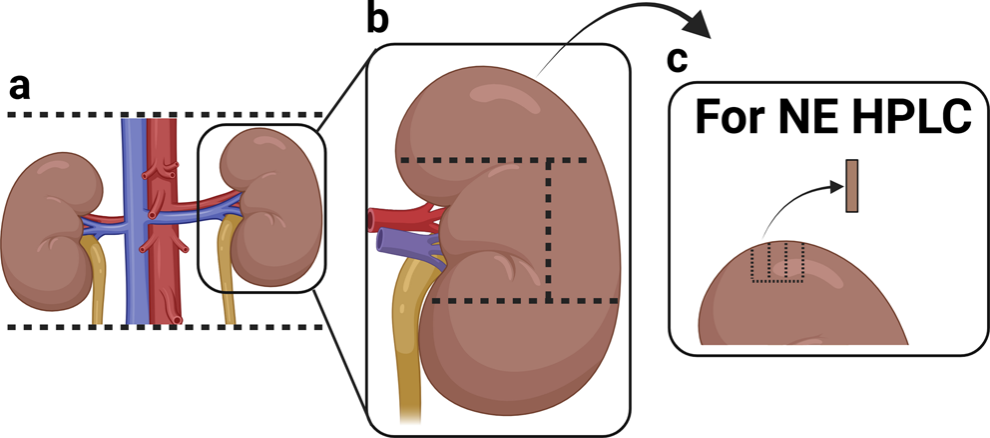
Terminal tissue collection. The (**a**) kidneys, renal arteries, renal veins, ureters, abdominal aorta, and inferior vena cava are removed from the sheep during tissue collection. The cephalad and caudal poles are (**b**) cut from the kidney, and (**c**) cortical slices are collected for Norepinephrine (NE) content measurement via high performance liquid chromatograph (HPLC). Created with BioRender.com.

Sheep were restrained before 300 IU Heparin was injected into the jugular vein to prevent coagulation. After allowing 1–3 min for the heparin to circulate, 87–90 mg/kg Euthasol (Virbac, NDC 051311-050-01) was injected into the jugular vein. After 1–2 min, the absence of spontaneous breathing and the corneal reflex was used to verify euthanasia before tissue collection. Both kidneys and the connecting segments of the renal arteries, renal veins, abdominal aorta, and inferior vena cava were harvested and rinsed twice in ice-cold normal saline to remove blood. The superior and inferior poles of each kidney were removed, and three separate 2 g slices of renal cortex were collected from each pole and flash frozen in liquid nitrogen before being stored at -80˚C for measurement of norepinephrine content by high performance liquid chromatography (NE HPLC). The lateral border of each kidney was removed to expose the renal pelvis. The remaining cortex, renal pelvis, renal hilum, and connecting vessels were rinsed with ice cold phosphate-buffered saline (PBS) and stored in Lana’s fixative (4% paraformaldehyde, 0.2% picric acid, 0.4 M phosphate buffer, pH 6.9) at room temperature for immunohistochemical (IHC) validation of denervation treatments (Fig. 2**)**.

### Confirmation of Denervation Efficacy: NE HPLC and CUBIC Tissue Clearing and IHC Staining

Renal cortical norepinephrine (NE) content was measured by running protein homogenates through high performance liquid chromatography with electrochemical detection (HPLC) to verify efferent renal nerve ablation [16,17,19,26].

**Fig. 3:**
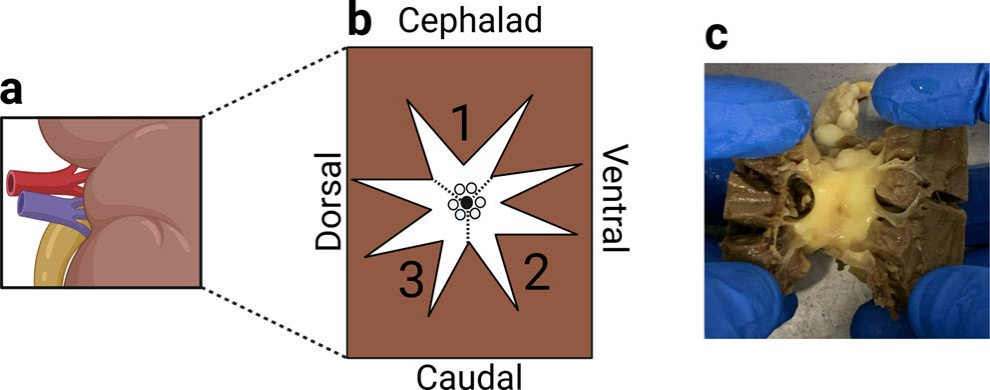
Renal pelvis tissue punch collection. The (**a**) renal pelvis, artery, vein, and ureter tissue block from Fig. 2 is fixed for 72 h in Lana’s fixative before being washed three times with ice-cold PBS. After the tissue has been washed, it can be stored in PBS at 4˚C until tissue punch collection. During tissue punch collection, remove excess renal cortex and medulla from the lateral aspect of the tissue block to expose the (**b**) renal pelvis and the ureteric opening. 6 mm tissue punches are then collected from the cephalad, ventral-caudal, and dorsal-caudal portions of the renal pelvis. Excess renal cortex or medulla are removed from the renal pelvis punches. Reference tissue image of exposed renal pelvis (**c**). Created with BioRender.com.

Tissue was cleared and then prepared for immunohistochemical analysis for the presence of efferent (tyrosine hydroxylase: TH) and afferent (calcitonin gene-related peptide: CGRP) renal nerves. Fresh Lana’s fixative solution was exchanged into the container containing the renal tissues, and vasculature 10 min, 24 h, and 48 h after tissue collection. After 72 h of tissue fixation, the tissues were washed with ice-cold PBS three times before storage in PBS at 4˚C. At time of tissue processing, 6 mm diameter (~ 2–5 mm in height) tissue punches were taken for tissue clearing and IHC staining (Fig. 3).

Two tissue punches were collected from the cephalad, ventral, and dorsal pelvic regions bilateral to the lumen of the renal pelvis from each kidney. All tissue punches were then processed using a modified clear, unobstructed brain imaging cocktails and computational analysis (CUBIC) clearing method for mouse whole organs [30,31]. Tissue clearing using the CUBIC method allows for imaging through the entire depth of the tissue punch and analysis of nerve fiber density within the renal pelvis. Tissues underwent 3 PBS washes, each > 2 h, at room temperature (RT) on an orbital shaker, followed by overnight pretreatment with 50% CUBIC-L + buffer (TCI Chemicals, Catalog #T3740) diluted with deionized H_2_0 (diH_2_O) in a 37˚C water bath. Tissues were then delipidated with 100% CUBIC-L + buffer in a 37˚C water bath for 3 days, and then washed in PBS 3 times for > 2 h each at RT on an orbital shaker. Tissues were incubated in blocking buffer (0.03% Triton, 1% bovine serum albumin, 1% normal donkey serum, 0.01% sodium azide in PBS) overnight at RT on an orbital shaker before primary antibody staining. One tissue punch from each region was either stained for sympathetic efferent (Rabbit anti-tyrosine hydroxylase (TH), 1:300, EMD Millipore, AB152) or afferent (Rabbit anti-calcitonin-related gene peptide (CGRP), 1:300, Immunostar, Catalog #24112) renal nerve fibers to validate total and afferent renal denervation. Note two tissue punches from each region of the renal pelvis were required for IHC due to the limitation of host species for TH and CGRP primary antibodies that resulted in clear labeling of sheep kidney tissue. Primary antibody staining was performed overnight at RT on an orbital shaker. After primary antibody staining, tissues were washed 3 times, each > 2 h with PBS at RT on an orbital shaker. Both TH and CGRP labeled tissue punches were stained with a donkey anti-rabbit Cy3, 1:150 (JacksonImmuno, Catalog #711-165-152) secondary antibody overnight. After staining, tissues underwent 3 washes for > 2 h with PBS at RT on an orbital shaker and were pretreated with 50% CUBIC-R + buffer (TCI Chemicals, Catalog #T3741) diluted with diH_2_0 overnight. Tissues were stored in 100% CUBIC R + for refractory index matching before imaging.

### IHC Imaging

Tissue punches were placed in the middle of homemade silicone gaskets (approx. 25 × 25 × 1 mm, hole diameter approx. 8 mm, stacked for varying thicknesses) on a glass slide (VWR Vistavision microscope slides, Catalog #16004-368) and topped with a glass coverslip (Epredia coverslips, Catalog #52222). All tissue punches were oriented with the luminal side of the renal pelvis towards the glass coverslip and submerged in CUBIC R + buffer. Tissues were imaged on a Nikon A1Rsi HD confocal microscope with SIM super resolution fitted with a Nikon Ti-E motorized stage, and Nikon PlanApo DIC N1 10x dry-immersion/NA0.45 objective and controlled by NIS Elements 5.1 software. Fluorescent images were collected using 561 nm (Cy3) excitation laser with collected emissions ranging from 575 to 625 nm. Represntative images in Fig. 5 were adjusted for brightness, contrast, and color using FIJI to enhance viewing [32].

**Fig. 4:**
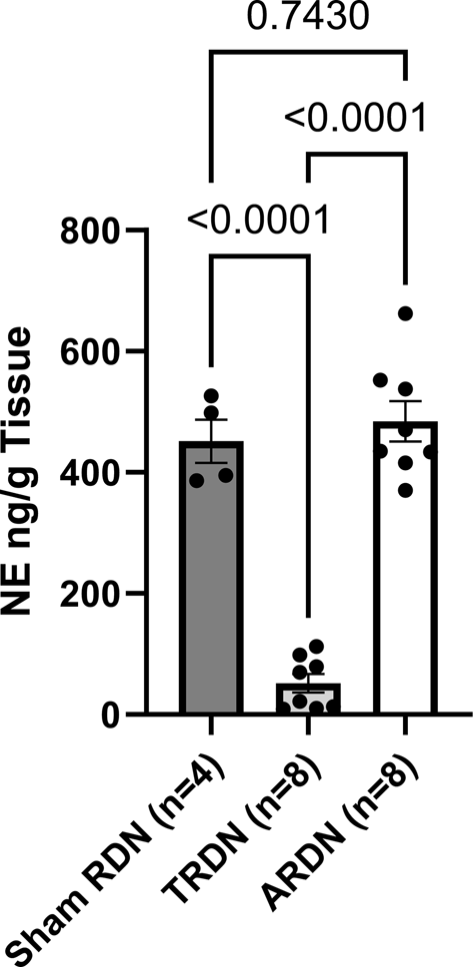
NE verification of denervation efficacy. TRDN (*n*=8 kidneys, 4 animals) significantly reduced the cortical NE content in comparison to the Sham RDN kidneys (*n*=4 kidneys, 2 animals). ARDN (*n*=8 kidneys, 4 animals) did not cause a significant decrease in cortical NE content in comparison to Sham RDN kidneys. Made with GraphPad Prism 10

**Fig. 5:**
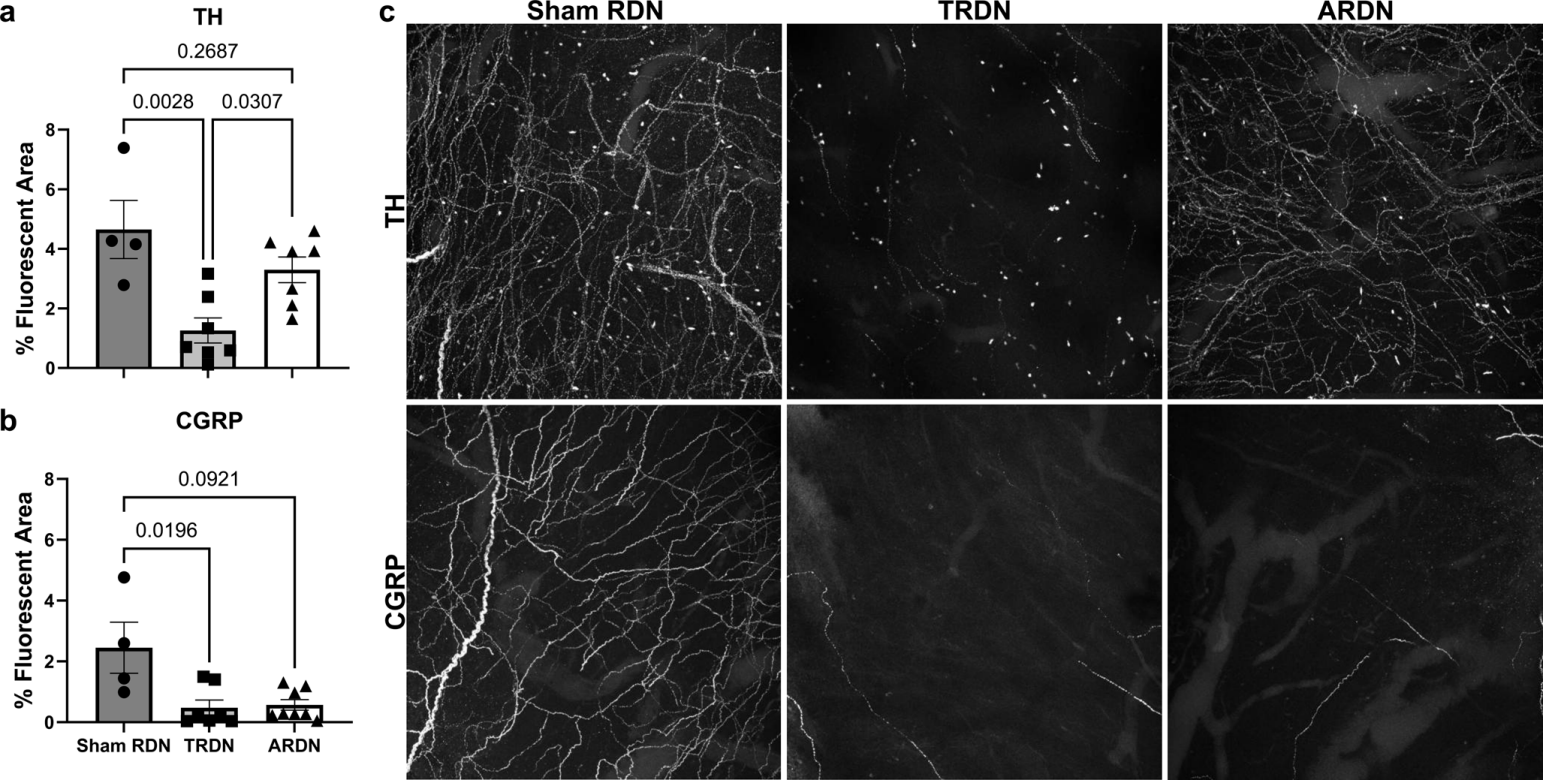
IHC verification of denervation efficacy. Average fiber density as a measure of percent fluorescent area of TH+ (**a**) and CGRP+ (**b**) fibers within the kidneys of Sham RDN (*n* = 4 kidneys, 2 animals), TRDN, and ARDN animals (both *n* = 8 kidneys, 4 animals). Fiber density was calculated by averaging percent fluorescent area across three fields of view in each tissue punch, averaged across tissue punches in one kidney. Each data point represents the average fiber density in one kidney. Error bars are mean ± SEM. P values above the brackets are from statistical analysis for (**a**) one-way ANOVA corrected for Tukey’s multiple comparisons test and (**b**) Kruskal-Wallis ANOVA corrected for Dunn’s multiple comparisons test. While there was a strong trend but no significant difference (*p* = 0.0921) between CGRP + fluorescence in Sham RDN and ARDN kidneys, a separate Mann-Whitney test showed ARDN kidneys had significantly lower CGRP + fluorescence than Sham RDN kidneys (*p* = 0.0162). Two outliers (outside two standard deviations from the mean) were removed in the TH analysis, one for TRDN and one for ARDN (Sham RDN *n* = 4, TRDN *n* = 7, ARDN *n* = 7 kidneys). One outlier was removed for the TRDN group in the CGRP analysis (Sham RDN *n* = 4, TRDN *n* = 7, ARDN *n* = 8 kidneys). (**c**) Representative images of TH + and CGRP + fibers from each group. Made with GraphPad Prism 10

Images of tissue punches were used to determine the density of the efferent and afferent renal fibers throughout the renal pelvis after TRDN, ARDN, or Sham RDN. Tissues were imaged in three fields of view each in a z-stack spanning 100 μm with a z-step of 5 μm, and one large image scan of the entire tissue punch with a focal point in the middle of the z-stack, e.g., 50 μm. Each z-stack from these three fields of view were opened in FIJI and processed into one image representing the maximum intensity projection of each z-step [26]. Each field of view was analyzed individually for the percent area fluorescence based on thresholding of the efferent (sympathetic, TH+) or afferent (sensory, CGRP+) fibers as a measure of fiber density. These values were then averaged across the three fields of view for the density of one tissue punch and averaged across tissue punches from the same kidney to get an average fiber density per kidney in each condition.

### Renal Artery Diameter Response To Renal Denervation Treatments

Fluoroscopic imaging was used to check for vessel leakage after ethanol (TRDN), capsaicin (ARDN), or saline (Sham RDN) injection. Renal artery diameter at the site of injection showed evidence of vasospasm, but the arterial diameter immediately distal to the site of injection either vasoconstricted or vasodilated based on the treatment. To quantify this, the renal artery diameter was measured ~ 0.5 cm distal to the site of injection before and at least 2 min after the first injection. Diametric calculations of fluoroscopic images were calibrated by using the outside diameter of the 7 F (2.33 mm) angiographic guide catheter. The percentage change in renal artery diameter was then averaged for each treatment group (TRDN, ARDN, and Sham RDN).

### Statistical Analysis

**Fig. 6:**
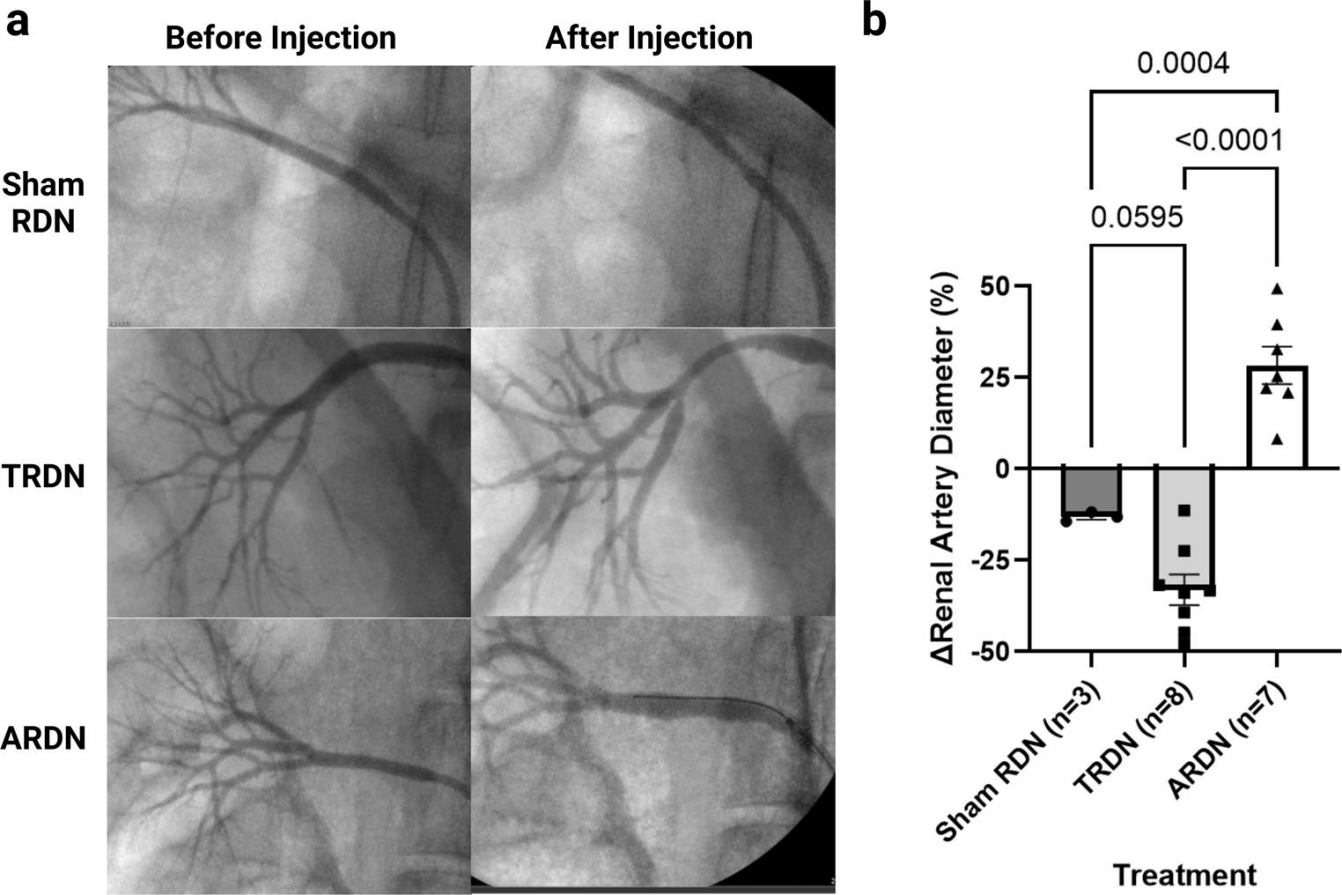
Renal artery diameter changes in response to TRDN, ARDN, and Sham RDN. Representative fluoroscopic images (**a**) of right renal arteries before and after injection of saline, ethanol, and capsaicin from the Peregrine™ catheter to perform Sham RDN, TRDN, and ARDN, respectively. Renal artery diameter measurements (**b**) were made from fluoroscopic images before injection and > 2 min after injection. Note the vasospasm after Sham RDN and TRDN, and vasodilation after ARDN. ARDN caused a significant increase in renal artery diameters in comparison to Sham RDN and TRDN. One outlier was identified in the Sham RDN and TRDN groups. Made with GraphPad Prism 10 and BioRender.com

All data was first tested for normality using the Shapiro-Wilk test. All data for renal cortical NE content, TH + IHC fluorescence, and renal artery diameter changes passed normality testing; therefore, one-way analysis of variance (ANOVA) and Tukey’s multiple comparisons test were used to compare treatment groups (Sham RDN, TRDN, and ARDN). The quantification of CGRP + IHC fluorescence failed to pass normality (Fig. 5b), so the Kruskal-Willis ANOVA with Dunn’s multiple comparisons test was used to compare Sham RDN versus TRDN or ARDN treatment groups. A Mann-Whitney test was then used to test for significant differences in CGRP + fluorescence between Sham RDN and ARDN. All analysis was computed with Graph Prism 10.2.2 and differences with a *p* < 0.05 were considered statistically significant. Results are reported as mean ± SEM. Statistical outliers were identified using Grubb’s test. One outlier was identified in the CGRP + IHC analysis of the TRDN group (alpha 0.05), and one outlier each was identified in the TH + IHC analysis of the ARDN and TRDN groups (alpha 0.1). Removal of these outliers is reflected in Fig. 5. One outlier was identified in the renal artery diameter measurements of the Sham RDN and TRDN groups (alpha 0.05). Removal of these outliers is reflected in Fig. 6. 

## Results

### Verification of Denervation Efficacy

TRDN significantly reduced renal cortical NE in comparison to Sham RDN (51.7 ± 13.4 vs. 451.5 ± 35.7 NE ng/g Tissue, *P* < 0.0001, Fig. 4). In contrast, ARDN had no significant effect on renal cortical NE content compared to Sham RDN (484.6 ± 29.4 vs. 451.5 ± 35.7 NE ng/g tissue, *P* = 0.7430, Fig. 4).

Figure 5 shows the results for quantification of the IHC analysis. Average percent fluorescent area per kidney in each group is shown in Fig. 5a-b with representative images of fiber density in Fig. 5c. One-way ANOVA with Tukey’s multiple comparisons test showed that TRDN caused a 72.9% decrease in TH + fiber density compared to Sham RDN (TRDN: 1.26 ± 0.42% vs. Sham RDN: 4.64 ± 0.97% *P* = 0.0028). ARDN did not cause a statistically significant decrease in TH + fiber density (ARDN: 3.29 ± 0.43% vs. Sham RDN: 4.64 ± 0.97%; *P* = 0.2687, Fig. 5a). Kruskal-Willis ANOVA with Dunn’s multiple comparisons test showed that TRDN caused a significant 80.3% decrease in CGRP + fiber density compared to Sham RDN (TRDN: 0.48 ± 0.25% vs. Sham RDN: 2.44 ± 0.85%, *P* = 0.0196, Fig. 5b). Initial analysis with a Kruskal-Willis test showed a trending 76.6% decrease in CGRP + fiber density between ARDN (0.57 ± 0.18% and Sham RDN (2.44 ± 0.85%, *P* = 0.0921). A two-tailed Mann-Whitney test showed that ARDN caused a significant decrease in CGRP + fiber density compared to Sham RDN (*P* = 0.0162).

### Catheter-Based ARDN Causes Transient Vasodilation of the Renal Artery

Fluoroscopic imaging was done before and after infusion of each solution to check for vascular leakage. One notable finding was the significant distal vasodilation (+ 28.3 ± 5.1%) that occurred shortly after the injection of capsaicin. This contrasts with the significant vasospasm following injection of ethanol or heparinized saline (TRDN: -33.1 ± 4.2% and Sham RDN: -13.1 ± 0.8%) (Fig. 6). One Sham RDN and ARDN kidney were excluded as outliers following Grubb’s test.

## Discussion

Catheter-based renal denervation is FDA-approved for the treatment of HTN [4,5] and is on the verge of widespread clinical practice. However, the mechanism underlying its efficacy is unknown. Preclinical studies in rodents from our lab [17,18,19] and others [20] have challenged the traditional view that the mechanism of action is due to ablation of the sympathetic renal nerves and have found a key role for sensory renal nerves in the development of HTN. The studies presented here represent the first application of afferent renal denervation (ARDN) in a large-animal model as well as the first catheter-based method for ARDN. To enhance the translatability of the findings and methods, sheep were used to enable the use of clinical trial catheters while retaining the anatomical, physiological, and hemodynamic similarities to humans [33]. More specifically, ovine kidneys have single renal arteries with similar anterior and posterior division proportional volume to human kidneys [34]. While no studies have effectively compared direct recordings of ovine versus human renal nerve activity, a previous study showcased the anatomical and functional reinnervation of ovine kidneys after radiofrequency-based renal denervation [35]. In addition, a clinical study reported how sevoflurane anesthesia caused water and sodium retention (an effect of renal sympathetic nerve activity) in pediatric surgery that was replicated and further explored in conscious and anesthetized ewes [36,37]. An ovine model is also significantly more cost-effective than swine because of their relatively stable size and docile nature which allows researchers to easily perform repetitive biopsies as well as conscious blood or urine sample collection for animal welfare and additional experimental analyses. Furthermore, it is possible that ARDN will provide similar HTN control as TRDN, without the potential deleterious effects observed in sheep exposed to experimental hemorrhage and sepsis [21,22]. If future preclinical studies show that catheter-based ARDN can lower blood pressure to the same degree as catheter-based TRDN, then catheter-based ARDN would have a clinically relevant benefit over catheter-based TRDN if it is used to treat HTN patients in the future. Future studies of the antihypertensive effects of catheter-based ARDN could be accomplished using this technique and the well-established Deoxycorticosterone (DOCA)-salt induced hypertensive sheep model [38,39,40,41].

ARDN was performed on sheep using Ablative Solution’s Peregrine™ catheter. The Peregrine™ catheter is still undergoing clinical trials but has recently reached its phase III clinical trial’s primary endpoint by demonstrating a statistically significant difference in 24-hour ambulatory blood pressure three months after TRDN [6]. In this successful clinical trial, TRDN was performed through the infusion of ethanol into the renal artery adventitia. Here, we have demonstrated that this catheter can be repurposed to infuse capsaicin to achieve afferent denervation while leaving the sympathetic nerves intact. Immunostaining confirmed that both ARDN and TRDN significantly decreased CGRP + fibers in renal pelvic samples, indicating that both TRDN and ARDN successfully ablated the renal afferent nerves. Just as in our rodent studies, TRDN led to a 90% reduction in renal cortical norepinephrine (NE) levels while ARDN had no significant impact on NE, indicating a selective ablation of the renal afferent but not the renal efferent nerves. To our knowledge, this is the first demonstration of intra-arterial ARDN– all prior rodent studies have performed ARDN surgically through either a laparotomy or a retroperitoneal incision.

In addition to proving the feasibility of repurposing the Peregrine™ catheter to perform ARDN in a large animal model, this technique could potentially be translated to treat hypertensive patients in the clinic in the future. However, this treatment would be more effective if there was a biomarker that could be used to predict the blood pressure lowering effect of ARDN as well as TRDN. Our group is currently determining the potential of urinary cytokines and chemokines to serve as a biomarker for the blood pressure lowering effect of ARDN. Previous studies from our group have shown that DOCA-salt HTN in rodents causes renal inflammation that leads to increased renal and urinary cytokine/chemokine content [42]. Moreover, the increases in renal and urinary cytokines/chemokines parallel the increase in arterial pressure and are reduced in response to TRDN or ARDN [17,26,42]. Previous studies in pain research have already shown how inflammatory mediators can modulate sensory nerve transmission [43,44,45,46], so it is possible that afferent renal nerve activity could be modulated by renal inflammation and inflammatory cytokines. One clinical study reported that urinary TNFα correlated with blood pressure in patients with HTN and psoriasis or rheumatoid arthritis [47]. A related study then showed that TNFα inhibition with infliximab reduced 24 h ambulatory blood pressure in rheumatoid arthritis patients [48]. We have also recently published a preclinical study that suggests that IL-1 receptor activation leads to increased afferent renal nerve activity and hypertension in the DOCA-salt mouse model [18]. Furthermore, this technique of sensory nerve ablation and sheep model could also be used to target the sensory nerves of other organs such as the liver in which increased sensory nerve activity contributes to pathological conditions [49,50].

### Limitations

This study was initially a pilot study to evaluate the feasibility of performing catheter-based afferent renal denervation by repurposing the Peregrine™ catheter. Due to the initial nature of this study, the relative number of animals per group is low and stretches the statistical power that can be used to analyze our data. While the impact of the low animal number is a clear limitation of the study, the data presented here highlights the effectiveness of catheter-based afferent renal denervation and the need for additional cohorts. Expanding this study will also allow the comprehensive examination of the hemodynamic and other physiologic effects of catheter-based afferent renal denervation, which will provide insights into the translatability as a treatment for those with treatment-resistant hypertension.

## Data Availability

All data from this study is available upon reasonable request from the corresponding author, JWO.
